# Handling techniques and risk factors reported by veterinary professionals during dog examinations: a cross-sectional survey across Canada and the United States

**DOI:** 10.3389/fvets.2025.1634970

**Published:** 2025-08-18

**Authors:** Lindsay Nakonechny, Alissa Cisneros, Carly M Moody, Anastasia Chiara Stellato

**Affiliations:** 1 Department of Animal and Food Sciences, Texas Tech University, Lubbock, TX, United States; 2 Department of Animal Science, University of California, Davis, Davis, CA, United States

**Keywords:** physical restraint, routine examination, dog welfare, dog fear, dog aggression

## Abstract

**Introduction:**

Handling techniques are known to influence dog stress in veterinary settings; however, little is known about the current handling techniques applied to dogs during routine veterinary care or risk factors associated with their use. This cross-sectional survey aimed to assess common handling techniques used on calm, fearful, and aggressive dogs by veterinary professionals in Canada and the United States and identify risk factors for minimal and full-body restraint.

**Methods:**

A convenience sample of veterinary professionals completed an online questionnaire. It collected information on participant characteristics and clinic experience (e.g., gender, Ten Item Personality Index, bite history, stress-reducing certification), participant professional quality of life (using the ProQOL scale), general examination practices (e.g., use of treats), perceptions and importance of examination factors (e.g., staff safety), and frequency of using 14 different dog handling techniques. Logistic regression models were used to identify risk factors for the use of minimal and full-body restraint on fearful and aggressive dogs.

**Results:**

Participants (*N* = 691) were veterinarians (39.2%, 271/691) and non-veterinarians (60.8%, 420/691), who routinely handle dogs during routine examinations in Canada (21.7%, 150/691) and the United States (79.1%, 541/691). Minimal restraint was reported to be used for calm (82.7%, 566/684), fearful (73.1%, 499/683), and aggressive (51.9%, 352/678) dogs during routine examinations. Full-body restraint was commonly reported to be used for calm dogs (58.5%, 400/684) and most frequently reported for fearful (63.9%, 434/679) and aggressive dogs (68.6%, 465/678). Handling decisions were influenced by factors including age, gender, practice type, graduation year, bite history, stress-reducing certification, and owner presence. Professionals prioritizing staff safety and using stress-reducing strategies (e.g., treats) were more likely to use minimal restraint, while owner presence and focus on examination completeness were linked to full-body restraint. Personality traits and professional well-being, particularly extraversion and secondary traumatic stress, also influenced handling choices.

**Discussion:**

Handling techniques vary with dog behavior and are shaped by numerous factors, highlighting the complex relationship between personal and clinic-level influences on veterinary staff interactions with dog patients. These findings generate hypotheses for future observational research exploring factors that support stress-reducing techniques to improve dog welfare in clinical settings.

## Introduction

More than 74% of dogs in North America are reported to visit a veterinary clinic each year (1), yet these appointments can be a negative experience for many dogs (2–4). Negative experiences may lead to increased dog fear during future veterinary visits (4–6) which increases the risk of aggression, and may impact the ability to perform comprehensive examinations and diagnostic procedures (7). Experimental studies in dogs suggest that stress can activate physiological pathways that reduce healing (8, 9) and impair immune function (10). While evidence for veterinary-related stress and its impact on dog health is lacking, these findings raise concerns that repeated or high levels of stress during veterinary care may contribute to delayed recovery or poorer health outcomes in some patients. Displays of aggression also pose a safety risk to veterinary staff, as animal bite and scratch injuries are among the most common injuries reported by veterinary staff across various countries, including the United States (11–14). In a recent study, almost 50% of surveyed Australian veterinarians reported experiencing a dog bite within the past year (15). Dog bites may lead to serious injury, bite-related infections, and even mortality in veterinary practice (13). Recommendations to reduce dog fear and aggression include providing treats throughout the appointment to facilitate a positive association with the clinic and staff, conducting examinations on the ground with a traction surface (16, 17), and having an owner present (18). A recent study found that dogs examined with practices aimed at reducing dog stress (e.g., treat provision, examinations on ground, use of a traction surface, minimal to no handling) had reduced serum cortisol levels and behavioral stress scores between their initial and final visits across four appointments, compared to the control group that received routine care (19, 20). Although this study involved a small sample size and its generalizability may be limited across clinical contexts, stress-reducing practices are recommended based on anecdotal evidence, and a growing body of research supports their use in veterinary care despite limited empirical data on their direct impact on dog stress.

There is interest in the use of handling techniques that minimize dog fear during veterinary care as the level and duration of physical restraint applied during examinations and procedures can elicit stress (16). As such, it is recommended to use stress-reducing techniques which involves making handling decisions based on dog behavior, starting with the least invasive restraint and increasing as needed to complete an examination or procedure (21–24). However, in the event of observed distress or aggression during an examination, it is recommended to pause or stop all handling, and/or reschedule the examination and develop a behavior modification strategy for the owner to implement between veterinary visits (17, 22–25). Applying behavior modification strategies, such as desensitization and counter-conditioning to veterinary-style handling, can help reduce the level of or need for physical restraint during routine veterinary care (16, 17, 22–25). In addition, pre-visit medications may also be prescribed to reduce dog stress and facilitate completion of an examination (17, 22, 24, 25). Dogs may also have certain areas of their body that are more sensitive to human handling, and different restraint techniques may result in varied fear responses. For instance, increased fear has been associated with head examinations, and palpation of a dog’s shoulders, paws, hind legs, and lymph nodes (18, 26, 27). Limited research exists on areas of the body that elicit positive behavioral and physiological responses in dogs; however, anecdotal recommendations suggest that the chest or under the chin may be more positively received during handling or other interactions, such as petting (22, 28). Dog responses to handling may be influenced by factors, such as breed, medical conditions, age, previous handling experiences, and clinical context (e.g., routine care vs. emergency treatment), and further research is needed to explore responses to handling of certain body areas while considering these individual and contextual factors. Full-body restraint (i.e., placing the animal in lateral recumbency with the front and hind legs held) has been associated with negative physiological and behavioral responses in dogs and other companion animal species (24, 29–31). A recent investigation of canine behavioral responses to common veterinary handling techniques suggests that dogs vocalize more during full-body restraint than minimal restraint, and that fear scores increase with the level of restraint; minimal restraint results in the lowest fear scores and full-body restraint the highest (32).

Although stress-reducing handling techniques are recommended, there are many factors that may influence handling decisions. For example, the type of veterinary practice may influence the types and frequency of handling techniques used. Veterinary professionals in emergency clinics frequently encounter injured or ill dogs, where pain-related aggression and fear responses (33, 34) may be higher, and prompt staff to apply higher levels of restraint to ensure quick diagnoses, and treatment. In these situations, higher levels of restraint may be necessary to provide urgent care. Handling decisions may also be shaped by dog characteristics, as factors such as demeanor (e.g., calm, fearful, aggressive), size, age, breed, behavioral history, and medical or pain status can influence perceived risk and physical demands of handling (3, 5, 18, 26). For example, patients with known histories of aggression or those experiencing pain may prompt heightened caution, which may result in the application of techniques that prioritize safety. These techniques may involve more or less restraint, depending on their previous training and education (e.g., stress-reducing certification, veterinary curriculum), clinical experiences, and clinic policies (e.g., use of muzzles). Perceptions and prioritization of certain examination factors could potentially influence animal handling. For instance, a survey of veterinary students and veterinarian attitudes found that the biggest perceived barrier to implementing stress-reducing techniques was time constraints, with the use of minimal restraint and other stress-reducing practices (e.g., examine the animal where they are comfortable or having the dog owner present) received lower ratings of importance and feasibility from students (35). Thus, it is possible that factors such as individual age, graduation year, and prioritization of certain factors (e.g., time to complete examinations over animal stress) may affect the perceptions and use of certain handling techniques by veterinary professionals.

Factors related to veterinary professionals are also hypothesized to influence handling techniques used during routine examinations. For instance, veterinarians have been reported to use minimal restraint techniques on cats less frequently compared to non-veterinarians (36) and having a stress-reducing certification (e.g., Fear Free for Veterinary Professionals) has been associated with more frequent use of minimal restraint techniques on dogs, compared to those without certification (4). Also, veterinary professional mental health and well-being may influence handling, as a qualitative interview study of Canadian veterinarians suggests that veterinarians indicated that high stress and poor mental health negatively impact their provision of care, such as shortening an examination and not providing animal patients with extra attention (37). The Professional Quality of Life (ProQOL) scale is a measure of well-being that is specific to individuals who work in helping professions (38) including veterinary professionals, as they provide help and care for animal patients and their owners. ProQOL assesses both negative and positive aspects of work-related experiences, including burnout, secondary traumatic stress, and compassion satisfaction (38). To date, the well-being of veterinarians has been examined for its impact on client satisfaction (39), but not for its impact on animal patients. In livestock research, stockperson well-being and stress levels have been reported to impact livestock interactions, with high stress associated with unwillingness to use proper handling practices (40) and greater well-being associated with positive animal welfare indicators (41–44). The reported relationship between human well-being and animal handling, along with research on veterinarian mental health affecting care provision, suggest these factors may also influence companion animal handling in veterinary settings.

Despite the growing interest and knowledge in ways to support dog welfare during routine veterinary care, including stress-reducing techniques, little is known about the handling methods currently used by veterinary professionals, as well as factors that influence handling decisions. Handling techniques used during routine examinations are likely influenced by multiple factors, including the veterinary staff, the dog patient, the clinic, and examination practices. Thus, the objective of this study was to identify the handling techniques used by veterinary staff for calm, fearful, and aggressive dogs, and identify factors associated with the reported use of minimal (lower restriction) and full-body (higher restriction) restraint during routine examinations.

## Methods

This cross-sectional survey was reviewed and approved by the Texas Tech University Research Ethics Board (#IRB2023-462) for research involving human participants.

### Data collection

An online cross-sectional questionnaire was distributed to veterinary professionals who handle dogs routinely at veterinary practices across Canada and the United States. Inclusion criteria for participants required individuals be 18 years of age or older, a current resident of Canada or the United States, and an actively working licensed veterinarian, or veterinary staff member (either licensed or unlicensed) who handles dogs during routine health examinations. We used convenience, snowball sampling via advertisements distributed on social media. This sampling technique has been demonstrated to reach targeted populations who are challenging to access (45), such as veterinary professionals. Email invitations were also sent to various Canadian and American veterinary organizations (e.g., Canadian and American Veterinary Medical Associations). The questionnaire was advertised and available from October 2023 to February 2024. Participation was voluntary and anonymous, and participants were provided with informed consent before accessing the questionnaire. The questionnaire took approximately 15 min to complete. Participant responses were not connected to any identifying information to minimize potential social desirability bias.

### Questionnaire

The online questionnaire was created using Qualtrics survey software (Qualtrics LLC, Provo, UT, United States). The questionnaire was comprised of 26 questions, categorized into five sections: (1) participant information and clinic experience: gender, age, graduation year, Ten Item Personality Index, staff role (veterinarian, non-veterinarian), practice type (small animal, mixed animal, emergency clinic), dog bite history; (2) participant professional quality of life: using the ProQOL scale; (3) general examination practices: provision of treats, stress-reducing handling certification, exam location, dog approach and handling upon entering the examination room, and response to dog struggle during restraint, (4) perception of whether certain factors influence handling of dogs during a routine examination and ranked importance of certain examination factors; and (5) frequency of using 14 dog handling techniques and tools. For the full list of questions provided in the questionnaire, see the Supplementary material.

The Professional Quality of Life (ProQOL) scale was used to measure participants’ subscale scores of compassion satisfaction (CS), burnout (BO), and secondary traumatic stress (STS). Each subscale includes 10 items with a 5-point Likert scale (1 = never; 5 = very often; 40). The scale was adapted to reflect the work of veterinary professionals. For example, “I find it difficult to separate my personal life from my life as a helper” was adapted to “I find it difficult to separate my personal life from my life as a care provider.” The ProQOL scale is a widely used validated measure of the positive and negative aspects of working with people who have experienced stressful or traumatic events (38), such as veterinary professionals. Personality traits of participants were measured using the Ten Item Personality Inventory (TIPI). This is a 10-item measure of the Big Five personality dimensions (Extraversion, Agreeableness, Conscientiousness, Emotional Stability, and Openness), including 2-items for each dimension, and each item is assessed on a 7-point scale (1 = disagree, 7 = strongly agree; 48). Participants were also asked to rate their level of agreement on whether the following factors influenced their handling of dogs during a routine examination: veterinarian instruction, patient behavioral history, dog breed, size, and age, owner presence, staff comfort level, and time to complete the appointment. They were also asked to rank the following five factors in order of importance (1 = most important, 5 = least important) when conducting a routine dog examination: completing the appointment on time, client satisfaction, staff safety, minimizing stress for dog patients, and completing all examination components.

The frequency of using 14 dog-handling techniques were assessed for small (under 35 lbs) and large (35 lbs. or greater) dogs using a Likert-scale (always, often, sometimes, rarely, or never). Participants were asked to report their frequency of using each handling technique if the dog was calm (patient is relaxed, no signs of aggression or fear-related behaviors), fearful (patient shows fear-related behaviors, such as lowered posture, ears back, tail tucked, whimpering or whining, shaking or trembling, attempts to hide or escape), and aggressive (patient showing fear-related behaviors while showing aggression, such as baring teeth, attempting to bite, growling, lunging). Participants were asked to separately report their responses for small and large dogs, as dog size was hypothesized to influence the choice of handling techniques used and some restraints include minor adjustments to accommodate dog size. For example, the image depicting full-body restraint included two handlers, compared to one handler for a small dog (21). The 14 handling techniques (including the written descriptions and images) used in this questionnaire were based on previous research by Carroll et al. (21) exploring dog owner perceptions of handling techniques used during routine examinations. The types of restraints assessed range from full-body restraint (i.e., placing the dog in lateral recumbency with the front and hind legs held), secure restraint (i.e., holding the dog’s abdomen securely with one hand and neck with the other hand), minimal restraint (i.e., hands are placed on each side of a dog’s shoulder allowing some movement of the dog’s body and limbs), and chemical restraint (i.e., sedative or anesthesia). The use of different restraint equipment was also assessed, such as basket and soft muzzles, dog masks (i.e., mask placed over the dog’s eyes and clipped behind the head to reduce visual stimulation), and Elizabethan collars (i.e., cone shaped collar that reduces head movement and mouth access). For full details on the 14 different handling techniques assessed for each dog demeanor, refer to the questionnaire found in Supplementary material.

### Statistical analysis

All analyses were performed with Stata Statistical Software v.15.1 (StataCorp, College Station, TX, United States).

### Data management

A total of 31 variables were used for analysis. Variables were related to participant information and clinic experience (12 variables), ProQOL (3 variables), general examination practices (3 variables), and perceptions and ranking of importance of certain examination factors (13 variables). Referent categories for categorical variables were selected based on the most common response, or biological plausibility specific to each variable. For instance, for staff role, the biological plausible referent chosen was non-veterinarians, which was more common than veterinarians. To reduce the number of variables tested for analysis, related variables were collapsed into composite variables. For example, the variable “non-veterinarian” was created from the following variables: veterinary technician (licensed), veterinary technician (non-licensed), and veterinary assistant. As these variables were all directly linked to one survey question regarding staff role, these variables were highly conceptually related and therefore a reliability analysis was not deemed necessary. Variables assessed with a Likert-scale of agreeance (strongly agree, agree, disagree, strongly disagree) were collapsed into categories of “agree” and “disagree” for ease of interpretation. Though dichotimzation of this variable can reduce the sensitivy of models to detect variation in frequency of handling technique use, this approach aligned with our aim to evaluate factors associated with the use (vs. non-use) of minimal and full-body restraint and has been applied in similar cross-sectional studies (36). During data cleaning, questions with “other” options were cross-referenced with existing options to reduce misclassification bias. Responses suspected of being from automated bots were identified and removed based on speed of survey completion and open-text anomalies, such as non-sensical text answers (46, 47). Further, in efforts to reduce the risk of over-fitting, categories with very low response counts were collapsed or removed. Despite these efforts, these analyses should be considered exploratory, and the findings will require validation in future studies with larger samples.

### Risk factor analysis

The frequency of using minimal (lowest level of restraint) and full-body (highest degree of physical restraint and aversive for dogs) were selected as they represent disparate degrees of restraint (29, 32). To appropriately conduct logistic regression, the frequency of using minimal and full-body restraint were consolidated to create a binary outcome with two levels, “used” (always, often, sometimes) and “not used” (rarely, never). Participants reported their frequency of using each technique separately for small and large dogs, so models were created for each dog size. This resulted in a total of eight logistic regression models to assess the impact of the explanatory variables on the following outcomes: (1) use of minimal restraint during examination of fearful dogs, (2) use of minimal restraint during examination of aggressive dogs, (3) use of full-body restraint during examination of fearful dogs, and (4) use of full-body restraint during examination of aggressive dogs.

Linear relationships between continuous independent variables (CS, BO, and STS scores, and TIPI scores) and the outcome variables (minimal and full-body restraint use) were assessed using locally weighted regression curves and testing significance of the quadratic term (48). The relationship between the continuous explanatory variables and dependent variables were neither linear nor quadratic, and therefore, these variables were categorized (48). ProQOL scores were categorized based on established scale cut-points by Stamm (38), as low (range cutpoint), medium (range cutpoint), or high (range cutpoint), and TIPI scores were categorized as either below or above population norms (Extraversion: 4.44, Agreeableness: 3.23; Conscientiousness: 5.40, Emotional Stability: 4.83, and Openness: 5.38; 48), as recommended by scale developers (49). Correlations between all explanatory variables were tested, and none were found to have a correlation coefficient of >70%. Univariate analysis was performed to test the association of each independent variable against each outcome variable. Independent variables with a liberal *p*-value of *p* ≤ 0.20 were retained in the model to allow potentially meaningful variables within the model (48). Each multivariable model was then built using a forward stepwise selection process where only significant explanatory variables (*p* < 0.05) were retained in the full model. Confounders were based on biological plausibility and were identified as an explanatory variable that caused >30% change in the coefficient of another variable in the model. This threshold was chosen as a conservative criterion for identifying covariates with substantial influence on other variable coefficients (48). Biological plausibility was assessed on potential influence on handling decisions, informed by previous literature on individual characteristics and animal handling (36, 42). When confounders were identified they were included in the model to account for confounding bias. To achieve parsimony, only significant and biologically meaningful interactions were included if they resulted in lower Bayesian information criterion (BIC) values. BIC was used to determine the appropriate model for the given dataset. Results were reported as the odds ratios (OR), where OR greater than one indicates higher odds (possible causal effect) and OR less than one indicates lower odds (possible protective effect). With a pre-determined Type I error rate set at 5%, 95% confidence intervals were also reported for each finding. Given the cross-sectional design of this study, the term “risk factor” is used descriptively to refer to variables that are statistically associated with the use of minimal or full-body restraint. These associations do not imply causality.

## Results

### Participants

A total of 920 responses were collected from the questionnaire, and 691 complete responses were retained for analysis after removing 229 suspected bot responses (46) and non-qualifying participants based on eligibility criteria. Most participants resided in the United States (78.3%, 541/691), identified as female (62.2%, 429/690), and were 25–44 years of age (77.4%, 531/686). Participants were non-veterinarians (67.7%, 454/671) and veterinarians (32.3%, 217/671) and work at small animal practices (52.4%, 361/689), mixed animal practices (30.3%, 209/689), emergency clinics (15.2%, 105/689), and/or other, such as shelter medicine (2.0%, 14/689). Most participants reported having a stress-reducing certification (86.9%, 544/626), which included certifications (86.9%, 544/626), which included certifications such as Fear Free Certified Veterinary Professional, Sophia Yin Low Stress Handling^®^ Silver Certified, and Karen Pryor Better Veterinary Visits. For full details on participants refer to Supplementary Table 1.

### Dog-handling practices

**Figure 1:**
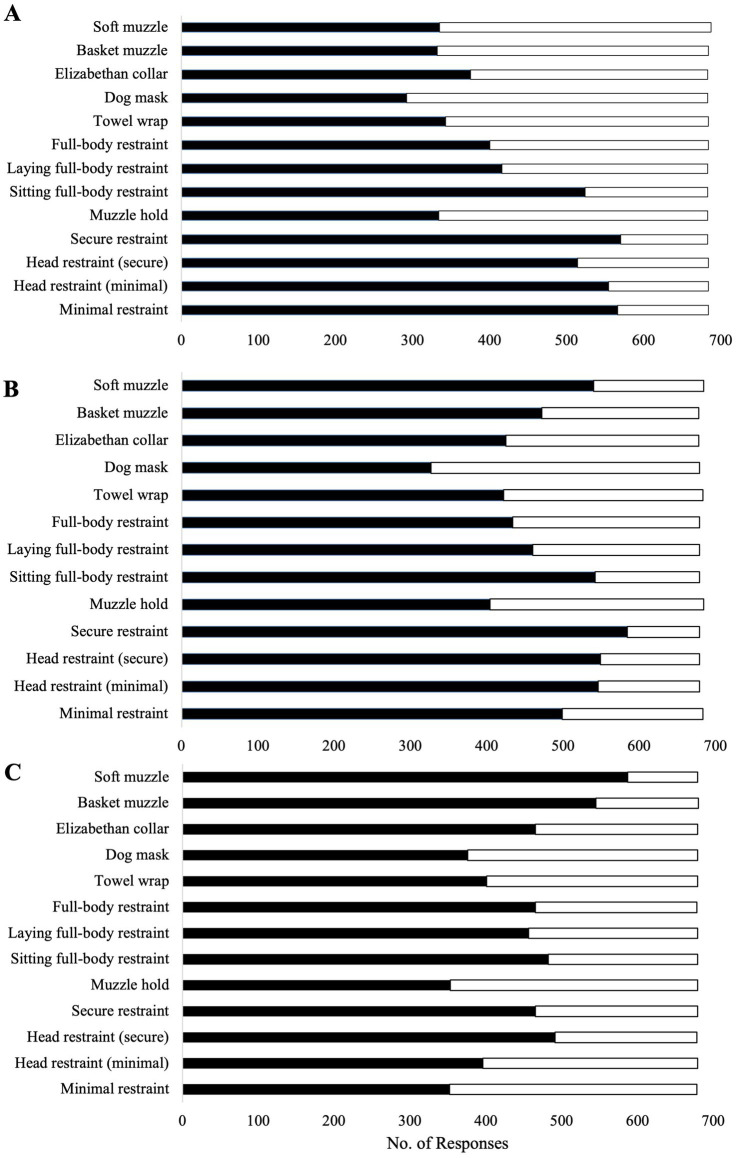
Reported frequency of use for 14 handling techniques and tools for calm **(A)**, fearful **(B)**, and aggressive **(C)** large dogs during routine examinations as reported by 691 veterinarians and non-veterinarians who completed an online questionnaire regarding dog-handling practices across Canada and the United States. The number of participants assessed for each technique varied, and no technique was assessed by all participants. Therefore, for each handling technique listed, the cumulative number of responses was <691. For the purpose of these charts, frequency of use was coded as always often, or sometimes (black bars), and rarely or never (white bars).

The frequencies of handling methods did not vary substantially by dog size. Therefore, only the frequencies for large dogs are reported, as this pattern was consistent across all explanatory variables in the descriptive data. Participants reported using minimal restraint most frequently for calm dogs (82.7%, 566/684), followed by fearful (73.1%, 499/683), and aggressive dogs (51.9%, 352/678; Figure 1). In contrast, full-body restraint was most commonly used for aggressive dogs (68.6%, 465/678), followed by fearful dogs (63.9%, 434/679), and calm dogs (58.5%, 400/684).

The general handling of dogs during routine veterinary examinations varied among participants (Figure 1). Minimal restraints were commonly reported for use on calm dogs; however, it was not uncommon for restrictive techniques (e.g., sitting full-body restraint) to also be reported. Fearful dogs were frequently reported to be handled using minimal and secure restraints, and for aggressive dogs, full-body and secure restraints were mostly used (Figure 1). Most participants use chemical sedation to restrain fearful (72.8%, 477/655) and aggressive (84.9%, 556/655) dogs. Participants indicated using various types of tools to restrain dogs of all demeanors, including dog masks (calm: 42.8%; aggressive: 55.4%) soft (calm: 48.8%; aggressive: 86.5%) and basket muzzles (calm: 48.5%; aggressive: 80.1%), and Elizabethan collars (calm: 54.9%; aggressive: 68.5%); however, all tools assessed were more frequently reported handling fearful and aggressive dogs and less for calm dogs. Complete data on the frequency of using the 14 handling methods on calm, fearful, and aggressive dogs is presented in Figure 1. When first entering an examination room, most participants reported that they allow the dog to explore the examination room before initiating handling (80.3%, 553/689). In addition, the majority indicated that they approach the dog indirectly (e.g., by crouching or kneeling and not facing the dog directly) rather than using a direct approach (e.g., standing or walking directly towards the dog; 19.0%, 130/685). Responses to a dog struggling during restraint varied. However, most participants reported that they release the restraint and allow the dog to calm down before reattempting (68.8%, 471/685). Less commonly, participants report either applying a more restrictive restraint or tool (15.6%, 107/685) or tightening their grip on the current restraint applied (15.6%, 107/685).

### Risk factors

Factors associated with the use of minimal and full-body restraint on small and large dogs during routine examinations were identified from logistic regression analyses and are reported in Tables 1–4.

**Table 1:** Final multivariable regression models for factors associated with minimal restraint use by veterinary professionals on fearful small (*n* = 658 participants) and large dogs (*n* = 639 participants) during routine examinations.

Model outcome	Variable	Category	OR (95% CI)	*P*-value
1. Use of minimal restraint during examination of fearful small dogs	Participant age	>44 years	Referent	–
		18–24 years	1.01 (0.50–2.05)	0.976
		25–34 years	1.83 (1.06–3.15)	**0.029**
		35–44 years	2.47 (1.35–4.52)	**0.003**
	Provision of treats	No	Referent	–
		Yes	2.20 (1.20–4.03)	**0.011**
2. Use of minimal restraint during examination of fearful large dogs	Participant age	>44 years	Referent	–
		18–24 years	1.46 (0.71–3.03)	0.306
		25–34 years	2.12 (1.22–3.68)	**0.008**
		35–44 years	2.18 (1.19–3.97)	**0.011**

Only variables with values of *P* < 0.05 were included in the final multivariable model for each outcome. — = not applicable. Bold values indicate statistical significance at *P* < 0.05.

**Table 2:** Final multivariable regression models for factors associated with minimal restraint use by veterinary professionals on aggressive small (*n* = 524 participants) and large dogs (*n* = 496 participants) during routine examinations.

Model outcome	Variable	Category	OR (95% CI)	*P-*value
1. Use of minimal restraint during examination of aggressive small dogs	Participant gender	Female	Referent	–
		Male	2.13 (1.40–3.24)	**<0.001**
	Participant dog bite history	No	Referent	–
		Unsure	5.61 (1.83–17.21)	**0.003**
		Yes	1.30 (0.85–1.99)	0.226
	Stress-reducing certification	No	Referent	–
		Yes	6.30 (2.94–13.47)	**<0.001**
	Influence of appointment time on dog handling	Agree	Referent	–
		Disagree	0.49 (0.32–0.76)	**0.001**
2. Use of minimal restraint during examination of aggressive large dogs	Participant gender	Female	Referent	–
		Male	2.64 (1.68–4.15)	**<0.001**
	Stress-reducing certification	No	Referent	–
		Yes	5.17 (2.30–11.61)	**<0.001**
	Practice type	Emergency clinic	Referent	–
		Mixed animal practice	2.44 (1.30–4.61)	**0.006**
		Small animal practice	1.02 (0.58–1.81)	0.942
	Exam location of large dogs	On the ground	Referent	–
		On a table	1.67 (1.05–2.66)	**0.036**
	Importance of completing all examination components	Rank 1 (most important)	Referent	–
		Rank 2	0.28 (0.12–0.63)	**0.002**
		Rank 3	0.36 (0.17–0.75)	**0.006**
		Rank 4	0.35 (0.17–0.71)	**0.004**
		Rank 5 (least important)	0.36 (0.18–0.69)	**0.002**

Bold values indicate statistical significance at *P* < 0.05.

**Table 3:** Final multivariable regression models for factors associated with full-body restraint use by veterinary professionals on fearful small (*n* = 543 participants) and large dogs (*n* = 466 participants) during routine examinations.

Model outcome	Variable	Category	OR (95% CI)	*P-*value
1. Use of full-body restraint during examination of fearful small dogs	Influence of owner presence on dog handling	Agree	Referent	–
		Disagree	0.65 (0.44–0.96)	**0.029**
	Importance of completing an appointment on time	Rank 1	Referent	–
		Rank 2	1.68 (0.85–3.33)	0.134
		Rank 3	2.15 (1.09–4.26)	**0.028**
		Rank 4	0.80 (0.45–1.44)	0.460
		Rank 5	0.57 (0.33–0.97)	**0.040**
	STS score	High	Referent	–
		Moderate	4.94 (1.36–17.89)	**0.015**
		Low	4.73 (1.37–16.33)	**0.014**
2. Use of full-body restraint during examination of fearful large dogs	Participant gender	Female	Referent	–
		Male	2.48 (1.51–4.07)	**<0.001**
	Graduation year	1971–1992	Referent	–
		1993–2003	0.48 (0.10–2.37)	0.367
		2004–2014	0.19 (0.04–0.94)	**0.041**
		2015–2023	0.43 (0.08–2.22)	0.313
		No degree	0.11 (0.02–0.71)	**0.021**
	Importance of staff safety	Rank 1 (most important)	Referent	–
		Rank 2	1.99 (1.07–3.69)	**0.030**
		Rank 3	2.50 (1.24–5.05)	**0.011**
		Rank 4	1.19 (0.56–2.52)	0.659
		Rank 5 (least important)	2.92 (1.39–6.11)	**0.005**
	Importance of completing all examination components	Rank 1 (most important)	Referent	–
		Rank 2	0.39 (0.18–0.87)	**0.022**
		Rank 3	1.83 (0.82–4.05)	0.139
		Rank 4	0.58 (0.27–1.24)	0.158
		Rank 5 (least important)	1.22 (0.59–2.51)	0.588

Bold values indicate statistical significance at *P* < 0.05.

**Table 4:** Final multivariable regression models for factors associated with full-body restraint use by veterinary professionals on aggressive small (*n* = 533 participants) and large dogs (*n* = 517 participants) during routine examinations.

Model outcome	Variable	Category	OR (95% CI)	*P-*value
1. Use of full-body restraint during examination of aggressive small dogs	Influence of owner presence on dog handling	Agree	Referent	–
		Disagree	0.51 (0.34–0.76)	**0.001**
	Participant dog bite history	No	Referent	–
		Unsure	1.76 (0.70–4.46)	0.230
		Yes	0.65 (0.42–0.99)	**0.048**
	Importance of staff safety	Rank 1 (most important)	Referent	–
		Rank 2	1.34 (0.75–2.41)	0.327
		Rank 3	1.07 (0.56–2.06)	0.830
		Rank 4	1.79 (0.84–3.81)	0.134
		Rank 5 (least important)	3.54 (1.75–7.17)	**<0.001**
	Importance of completing all examination components	Rank 1 (most important)	Referent	–
		Rank 2	0.37 (0.16–0.87)	**0.022**
		Rank 3	0.89 (0.41–1.93)	0.761
		Rank 4	0.75 (0.35–1.61)	0.459
		Rank 5 (least important)	1.29 (0.62–2.69)	0.494
	Practice type	Emergency clinic	Referent	–
		Mixed animal practice	2.66 (1.40–5.09)	**0.003**
		Small animal practice	1.07 (0.61–1.86)	0.821
2. Use of full-body restraint during examination of aggressive large dogs	Influence of owner presence on dog handling	Agree	Referent	–
		Disagree	0.49 (0.32–0.75)	**0.001**
	Importance of staff safety	Rank 1 (most important)	Referent	–
		Rank 2	1.61 (0.87–2.97)	0.126
		Rank 3	1.21 (0.62–2.36)	0.573
		Rank 4	1.89 (0.86–4.17)	0.114
		Rank 5 (least important)	3.31 (1.61–6.78)	**0.001**
	Staff role	Non-veterinarian	Referent	–
		Veterinarian	0.48 (0.30–0.76)	**0.002**
	Influence of dog age on dog handling	Agree	Referent	–
		Disagree	0.50 (0.31–0.80)	**0.004**
	Importance of completing all examination components	Rank 1 (most important)	Referent	–
		Rank 2	0.24 (0.10–0.56)	**0.001**
		Rank 3	0.77 (0.34–1.72)	0.521
		Rank 4	0.50 (0.23–1.09)	0.080
		Rank 5 (least important)	0.95 (0.46–1.97)	0.887
	TIPI extraversion	Above normal	Referent	–
		Below normal	0.59 (0.38–0.93)	**0.022**

Bold values indicate statistical significance at *P* < 0.05.

### Minimal restraint

#### Risk-factors associated with minimal restraint of fearful dogs

For small dogs, participants aged 25–44 years of age had higher odds of using minimal restraint, compared to those over 44 years (Table 1). Additionally, the provision of treats during examinations was associated with higher odds of using minimal restraint (Table 1). Staff role was identified as a confounder on participant age and was retained in the model.

For large dogs, participant age was the only significant predictor. Consistent with small dogs, participants aged 25–44 years of age had higher odds of using minimal restraint compared to those over 44 years (Table 1). The following variables were identified as confounders on participant age and were retained in the model: staff role, and personality traits (extraversion, agreeableness, conscientiousness, openness, and emotional stability).

#### Risk-factors associated with minimal restraint of aggressive dogs

For small dogs, participants who identified as male, unsure of their previous dog bite history, or held a stress-reducing certification, had higher odds of using minimal restraint (Table 2). Additionally, participants who disagreed that available appointment time influenced their handling decisions had lower odds of using minimal restraint (Table 2). The following variables were identified as confounders and retained in the model: staff role, clinic type, CS score, and personality traits (extraversion, conscientiousness, openness, emotional stability).

For large dogs, participants who identified as male, conducted examinations on a table (vs. on the ground), held a stress-reducing certification, or worked at a mixed animal practice had higher odds of using minimal restraint (Table 2). Additionally, participants indicating a high importance of completing all components of a physical examination had higher odds of using minimal restraint (Table 2). The following variables were identified as confounders and were retained in the model: participant age, staff role, ProQOL scores (CS, BO, and STS), and personality traits (extraversion, agreeableness, conscientiousness, openness, and emotional stability).

### Full-body restraint

#### Risk-factors associated with full-body restraint of fearful dogs

For small dogs, participants with low or moderate STS scores had higher odds of using full-body restraint (vs. high STS scores; Table 3). Participants disagreeing that the owner’s presence influences their handling decisions, had lower higher odds of using full-body restraint (Table 3). Also, participants who ranked the importance of completing an appointment on time as 3 or above had higher odds of using full-body restraint; however, participants who ranked it as least important had lower odds of using full-body restraint (Table 3). Personality traits (conscientiousness, openness) were identified as confounders and retained in the model.

For large dogs, participants identifying as male had higher odds of using full-body restraint (Table 3). Participants who graduated from their veterinary program between 2004–2024 or had no degree (e.g., unlicensed veterinary technicians) had lower odds of using full-body restraint (vs. participants who graduated from 1971–1992; Table 3). Regarding perceptions, participants who ranked the importance of staff safety or completing all examination components as high, had higher odds of using full-body restraint. Participant age, clinic type, and personality trait (extraversion) were identified as confounders and retained in the model.

#### Risk-factors associated with full-body restraint of aggressive dogs

For small dogs, participants who reported not having a previous bite history or worked at a mixed animal practice had higher odds of using full-body restraint (Table 4). Regarding perceptions, participants who disagreed that an owner’s presence influenced their handling decisions had lower odds of using full-body restraint (Table 4). Also, participants who ranked the importance of completing all examination components and staff safety as high had higher odds of using full-body restraint (Table 4). Participant STS score and personality traits (extraversion, agreeableness, conscientiousness, and openness) were identified as confounders and retained in the model.

For large dogs, participants who worked as veterinarians had lower odds of using full-body restraint (vs. non-veterinarians; Table 4). Extraversion was the only personality trait predictive of handling techniques, where participants with below normal extraversion had lower odds of using full-body restraint (vs. above normal; Table 4). Regarding perceptions, and consistent with small dogs, participants who disagreed that an owner’s presence and dog’s age influenced their handling decisions had lower odds of using full-body restraint (Table 4). Participants who ranked the importance of staff safety as high had lower odds of using full-body restraint (Table 4); while higher odds were associated with a high ranking of completing all examination components (Table 4). Participant age, gender, ProQOL scores (CS, STS), and personality traits (agreeableness, openness, and emotional stability) were identified as confounders and retained in the model.

## Discussion

Results from the cross-sectional survey suggest that handling techniques used by veterinary professionals in Canada and the United States depended on the dog’s demeanor. Participants reported using minimal restraint more frequently with calm and fearful dogs, which is consistent with recommended practices for reducing dog stress in veterinary settings (22–25). However, participants reported using full-body restraint more often with fearful and aggressive dogs, contradicting stress-reducing principles that recommend pausing or stopping handling if aggression occurs. It is possible that participants also implemented alternative recommended strategies, such as pre-visit medications, behavioral modification or referral in conjunction with full-body restraint, but still deemed full-body restraint necessary to apply. These findings are similar to results from a veterinary cat handling survey, which suggests full-body restraint was used more often for fearful and aggressive cats during examinations, compared to calm cats (36). Veterinary professionals’ use of full-body restraint may reflect efforts to ensure staff safety and examination efficiency through increased control of a dog’s movement, despite its association with negative dog physiological and fear responses (24, 29–32). For instance, a study found that dog owners with previous veterinary experience reported higher agreement with the use of full-body restraint on fearful dogs (21). However, the perception of enhanced safety through increased restraint may be counterintuitive, and rather, may elicit and/or worsen dog fear and aggressive responses to avoid or escape the aversive restraint (16, 22, 24, 25).

Participants reported using chemical restraint (i.e., anxiolytics such as trazodone) during examinations for all dog demeanors, though mostly for fearful or aggressive dogs. This suggests that chemical restraint is used reactively, in response to dogs showing fear or aggression, as well as proactively, to prevent calm dogs from becoming fearful or aggressive during handling. These results align with recommendations, as administering chemical restraint may reduce stress and facilitate handling without inhibiting learning (50, 51), especially if paired with minimal restraint to support positive handling experiences (16, 17, 24). The types of handling applied with chemical restraint are unknown in this study; thus, future research should explore when chemical restraint is administrated and what handling methods are applied during sedation. Regarding handling tools, muzzles were the most frequently reported tools used for fearful and aggressive dogs, with soft muzzles reported more than basket muzzles. The use of soft muzzles may reflect a perception among veterinary professionals of greater safety, and they may be easier and quicker to apply. This contrasts with current recommendations for the use of basket over soft muzzles, as they do not restrict oral behaviors (e.g., painting, lip licking), breathing, or ability to consume treats (16, 24) and have been associated with lower fear scores in dogs during routine examinations (32). Although towel wraps are recommended as a less restrictive alternative to full-body restraint by providing control of the head (16, 17, 24), they were used less frequently than full-body restraint. While many veterinary professionals report using stress-reducing techniques, handling techniques and tools associated with increased arousal and stress in dogs (e.g., full-body restraint, soft muzzles) are still commonly used for dogs of all demeanors. Thus, future research should continue to explore these handling decisions and to promote continued education on the benefits associated with using stress-reducing techniques.

Although we detected some overlap in risk-factors for minimal and full-body restraint, differences across models suggest that handling decisions are multifaceted, highlighting the importance of using a comprehensive approach to examine factors that influence veterinary staff and dog patient interactions. For minimal restraint, primary predictors included participant characteristics (e.g., age, gender, practice type, having a bite history), general examination practices (e.g., provision of treats), and having a stress-reducing certification. Additional predictors reflected participants’ perceptions and priorities during examinations, such as the importance placed on staff safety and completing all examination components. For full-body restraint, predictors included professional quality of life (e.g., STS score) and differing perceptions of external influences, such as the presence of owners during examinations and the importance of staff safety.

### Staff safety

Handling decisions for aggressive dogs appear to be influenced by veterinary staff’s perceptions of safety and past experiences, including previous bite injuries. Participants who prioritized staff safety or had been bitten were less likely to use full-body restraint on fearful or aggressive dogs, and those unsure about having been bitten were more likely to use minimal restraint on aggressive dogs. In situations where the risk of injury is high, such as when dogs are aggressive, minimal restraint is advisable to avoid increasing arousal and aggression (24), thereby reducing the likelihood of injury. Although no research to date has directly examined the relationship between restraint level and injury risk, existing recommendations are primarily based on expert opinion and anecdotal evidence. Our findings suggest that prior experiences with injury may heighten risk perception among staff, leading them to adopt a more cautious approach, such as minimizing physical contact through the use of minimal restraint. While full-body restraint was frequently reported and may be perceived to enhance safety through increased control of movement, our results suggest that prior injuries may alter this perception and practice. Full-body restraint is associated with a greater likelihood of dog fear escalating to aggression (17, 22, 24, 25), and potentially, also higher risk of bite injury (23, 26). Experiencing a dog bite may lead veterinary professionals to recognize the association between high restraint and dog aggression, potentially motivating them to seek training in, or more consistently apply stress-reducing handling techniques. Further research should explore how factors, such as bite severity, frequency, antecedents, and the context of the injury shape risk perception, examination priorities (e.g., staff safety, appointment efficiency), and handling decisions.

### Perceptions and prioritization of examination factors

Participant perceptions of examination factors, such as completing all examination components and managing available time, also influenced dog handling practices. Specifically, staff who prioritized completing all examination components were more likely to use full-body restraint on large aggressive dogs but less likely to do so for small fearful and aggressive dogs. These findings may reflect the belief that full-body restraint is more appropriate or practical for handling aggressive large dogs due to their size, whereas less restrictive restraint may be perceived as sufficient for small dogs. Also, those who did not perceive available time as an important factor influencing handling decisions, were less likely to use full-body restraint on fearful dogs. This may suggest that without time constraints there is more flexibility in using less invasive techniques with fearful patients, whereas staff who feel rushed may rely on higher levels of restraint to adhere to a schedule and may avoid stress-reducing strategies they perceive to be inefficient and/or prolong appointments. This aligns with previous research that identified time constraints and high workloads as barriers to implementing stress-reducing practices in veterinary settings (4, 35). However, preliminary evidence suggests that these practices (e.g., minimal or no restraint) may not prolong appointments (20) and can improve ease of examination and promote positive experiences during visits (3, 5). These findings may not generalize to all practice settings and patients, and further research is needed to evaluate the time efficiency of stress-reducing approaches in a variety of real-world contexts. Moreover, while appointment structure and staffing support are important factors, we recognize that these strategies alone may not address the needs of all patients, particularly those with behavioral concerns or histories of aggression, where behavioral pharmacology and referral to behaviorists may be needed. Further, this warrants a need to explore how appointment structure and volume influences handling decisions. Education and training on effective and practical ways to implement stress-reducing strategies, catered to practice types (e.g., emergency, general practice) may also support the provision of comprehensive, efficient, safe, and lower stress veterinary care. Despite most participants holding stress-reducing certifications, barriers such as appointment structure, high workloads, and perceptions of efficiency may hinder the use of minimal handling in clinical practice, in some cases.

Owner presence during an examination also influenced handling decisions. Veterinary staff who felt that an owner’s presence did not impact their handling decisions were less likely to use full-body restraint on fearful and aggressive dogs, suggesting that they may feel less pressured by clients and more inclined to use less restrictive techniques and focus on other factors, such as the dog’s behavior. In contrast, those who report that owner presence impacts their handling decisions may use greater physical control of the dog (e.g., through full-body restraint) to ensure safety and comprehensive examinations; however, this approach conflicts with clients’ preferences. For instance, research shows that dog owners prefer minimal over full-body restraint, particularly if their dog is fearful (21, 52, 53), and they perceive full-body restraint as excessive, inappropriate, and stressful (52). Additionally, some research suggests that owner presence reduces behavioral signs of dog stress during examinations (18, 54), while others suggest owner presence may increase dog stress if owners have negative interactions with their dog during the appointment (e.g., verbal punishment or aversive handling), or if the owner is highly stressed during the appointment (17, 55, 56). It is possible, that veterinary staff who perceive or anticipate dog behavior to worsen with owner presence, may rely on higher levels of restraint. Future research is needed to continue exploring how factors like owner presence, staff priorities, appointment pressures, and safety concerns influence handling decisions to identify approaches that optimize safety, minimize dog stress, and align with client preferences while enabling comprehensive care.

### Participant characteristics

Having a stress-reducing certification and using recommended strategies for decreasing dog fear (e.g., provision of treats) were associated with increased use of minimal restraint on fearful and aggressive dogs, reflecting the application of stress-reducing principles (16, 17, 22, 24). A generational trend was also observed, with younger participants being more likely to use minimal restraint on fearful dogs, and those who graduated during the rise of Low-Stress Handling^®^ (22) being less likely to use full-body restraint on fearful dogs. This trend may reflect differences in curricula, as recent graduates or younger veterinary professionals may have had more education on stress-reducing handling principles and its benefits for staff and patients.

Participant gender also influenced handling decisions, where men were more likely to use minimal restraint on aggressive dogs. While our study did not assess underlying psychosocial variables, one possible explanation is that gender-related differences in risk perception may influence handling choices. Previous research has identified gender differences among veterinarians in areas such as client communication (57), and exposure to occupational stressors, such as high workloads (58). It is possible that women may perceive a higher risk of injury and opt for more restrictive handling when encountering dog aggression. However, this remains speculative and further research should directly examine how psychosocial factors, such as perceived risk, workload demands, performance pressures, work-life balance, and other systemic challenges shape handling decisions across genders.

Staff role and practice type influenced the use of full-body restraint, with increased use reported by support staff (e.g., technician or assistant). Veterinary technicians and assistants may use more restrictive handling potentially due to limited exposure to stress-reducing practices during their education/training or as a result of veterinarians delegating certain handling techniques to support staff. Regarding practice type, participants working in emergency clinics were less likely to use minimal restraint on aggressive large dogs. Emergency staff may be less likely to implement minimal restraint due to frequent exposure to highly distressed and possibly aggressive dogs and thus may opt for restraints that allow for more control of the dog’s movement to apply necessary treatment. However, emergency staff were also less likely to use full-body restraint on aggressive small dogs, which may suggest that handling techniques used in emergency settings may depend on dog size, potentially due to perceived risk differences (35). We did not examine differences in the implementation of stress-reducing techniques across practice types. Thus, future research should explore how contextual factors shape handling decisions in different clinical environments.

### Professional well-being and personality

Personality traits and professional quality of life (ProQOL) influenced the use of full-body restraint. Specifically, the use of full-body restraint on fearful dogs was more common among participants with low to moderate secondary traumatic stress (STS), than those with high STS. Individuals with high STS are typically overwhelmed by a negative experience at work, characterized by fear, and may experience symptoms such as exhaustion, sleep disturbances, and avoiding activities that are traumatic triggers (38). Thus, veterinary professionals who experience high STS may be more cautious about exposing themselves to potentially stressful situations or witnessing their patients in distress, such as dog fear or aggression that can result from full-body restraint (32), which is anecdotally physically and emotionally demanding on handlers. This aligns with recommendations for countering STS, which includes changing caseload, work environments, or introducing other safety measures (38). Thus, these individuals may avoid practices that induce fear for themselves or their dog patients as a way to cope or protect against further stress. In contrast, those with low or moderate STS may not feel the need to avoid such stressors when making handling decisions regardless of the potential stress elicited. Burnout and compassion satisfaction were not associated with handling practices, possibly due to limited variability in ProQOL scores. Alternatively, handling practices may remain unaffected despite experiencing BO or STS, due to unmeasured factors such as resiliency. or the ability to cope with stress (59). Resilience may enable professionals to manage their responses in stressful situations, such as when handling an aggressive patient. Similarly, Perret et al. (39) found no relationship between poor veterinarian mental health and low client satisfaction, suggesting client interactions may remain consistent even under psychological strain. Compassion satisfaction can help meditate compassion fatigue (60); thus, any effects that compassion fatigue (i.e., BO and/or STS) may have on patient interactions may be mitigated by having high satisfaction from their work. Categorizing veterinary professionals based on combinations of their ProQOL scores and examining these scores with handling practices (e.g., frequency of using minimal and full-body restraint) may capture other trends in animal handling.

Regarding the personality traits assessed, participants with lower extraversion scores were less likely to use full-body restraint on aggressive dogs. As extraversion reflects an individual’s sociability and positive emotionality, such as enthusiasm (61), results may suggest that less extraverted individuals may experience greater discomfort when applying higher levels of physical restraint. The relationship between personality, well-being, and patient handling is likely complex, and though the current study provides preliminary insights, more research is needed to further explore the role of these factors in shaping handling decisions.

## Limitations

The online questionnaire was distributed by email and social media, and thus our data was likely influenced by selection bias. For instance, our participants reflect individuals with an active email or social media account, and those familiar with accessing and using online surveys. The majority of participants graduated from a veterinary program between 2004–2023 and were between the ages of 25–44, which reflects the trend observed in web-based surveys of receiving greater responses from younger individuals (62). Also, a significant proportion of participants reported that they had a stress-reducing certification. Although there is no information available on the proportion of veterinary professionals in Canada and the United States who have these certifications, it is likely that individuals with an interest in stress-reducing practices and dog welfare would be more inclined to respond to this type of survey, thus reflecting a voluntary response bias. Only 19.2% of surveyed veterinary professionals have some form of stress-reducing certification in Australia, which is likely inflated due to convenience sampling (4); thus, it is unlikely that the number of participants with stress-reducing certifications reported in this study are representative of the greater North American veterinary population. Despite these limitations, the demographic composition of our sample, being predominantly female, working in small or companion animal practice, and younger or recent graduates, closely aligns with the broader population of employed veterinary professionals in North America (63–66). Thus, it is possible that the reported level of minimal restraint, which is a stress-reducing practice, is inflated within this sample. Further, most participants in the current study reported moderate ProQOL scores; however, prior research suggests higher rates of BO and STS among veterinary professionals in North America (66–69). This raises the possibility of non-response bias, where individuals experiencing higher occupational stress may have been less likely to participate, potentially limiting our ability to explore the relationship between well-being and handling practices. Future studies using direct observation of handling techniques during routine exams may help validate self-reported findings and minimize potential reporting biases.

There is also potential for social desirability and recall bias, with participants possibly underreporting the use of aversive techniques (e.g., full-body restraint) and overreporting stress-reducing approaches (e.g., minimal restraint). Notably, despite most participants holding stress-reducing certifications, many still reported using full-body restraint on calm dogs. This may reflect misinterpretation of the questions, with some respondents reporting restraint use across various contexts (e.g., emergency care), rather than specifically during routine exams. Given the possibility of social desirability bias and an intention-behavior gap, where reported frequencies may not reflect actual clinical practice, the questionnaire was administered anonymously to help encourage honest responses. Additionally, common-method bias may be present, as both exposures and outcomes were collected within the same questionnaire, which can inflate observed associations (70).

Further, due to the cross-sectional nature of this study, causal inferences cannot be made between risk factors and handling techniques used by veterinary professionals. Also, additional factors, such as staffing, financial resources, or clinic policies, were not assessed that may impact the implementation of certain handling techniques and stress-reducing practices. We did not conduct penalized regression or internal validation due to sample size constraints, and some significant associations yielded wide confidence intervals, reflecting imprecision likely due to low event counts or response variability. In addition, this study tested multiple associations across several regression models, which may increase the risk of Type I error. As such, these findings should be interpreted as exploratory and hypothesis-generating, with future studies needed to replicate and validate these associations. Although research on veterinary handling is growing, much of the available evidence is based on observational studies or expert consensus, with few controlled trials. Empirical research is still needed to better understand how handling techniques and individual, dog, and clinic-level factors influence staff responses to fear or aggression during routine examinations.

## Conclusion

Findings from this cross-sectional study indicate that veterinary professionals in Canada and the United States commonly use minimal restraint when handling dogs during routine examinations, which aligns with current recommendations; however, full-body restraint is used on calm, fearful, and aggressive dogs, despite many participants having a stress-reducing certification. Factors related to participant demographics, the veterinary clinic, general examination practices, ProQOL and personality traits, and perceptions and prioritization of certain examination factors were associated with minimal and full-body restraint use. Further research is needed to explore the predictors identified in the present study, particularly those associated with the use of full-body restraint, as there is growing evidence to suggest this is a stressor for dogs. These factors include handling-related bite injuries, perceived risks to safety, staff role, practice type, and perceptions of owner presence during examinations. Additionally, exploring how clinic pressures, such as appointment structure, clinic culture and management, and examination completeness relate to the use of different handling techniques. Investigating veterinary populations with more variable ProQOL scores may also reveal further insights into how well-being relates to handling strategies. This study offers valuable insights into the various factors that influence handling decisions during routine examinations, which is essential for safeguarding dog health and welfare, as well as enhancing the experiences and safety of owners and veterinary staff.

## Data Availability

The raw data supporting the conclusions of this article will be made available by the authors, without undue reservation.
