# Correction: Handling techniques and risk factors reported by veterinary professionals during dog examinations: a cross-sectional survey across Canada and the United States

**DOI:** 10.3389/fvets.2026.1845949

**Published:** 2026-04-14

**Authors:** Frontiers Production Office

**Affiliations:** Frontiers Media SA, Lausanne, Switzerland

**Keywords:** physical restraint, routine examination, dog welfare, dog fear, dog aggression

Upon publication, Supplementary material “**Supplementary File 1**” was accidentally omitted. The file has now been published.

The original version of this article has been updated.

