# Supplementary material for: Handling techniques and risk factors reported by veterinary professionals during dog examinations: a cross-sectional survey across Canada and the United States
Source: Front Vet Sci. 2025 Aug 18;12:1634970. doi: 10.3389/fvets.2025.1634970 (PMC12400514; doi:10.3389/fvets.2025.1634970)
Supplement: Supplementary file 1 [file Table_1.docx]

Supplementary Material

**Table 1.** Summary statistics for 691 veterinary professionals who completed an online questionnaire regarding dog handling techniques during routine examinations in Canada and the United States.

| Variable | No. of respondents (%) |
| --- | --- |
| Age (years) |  |
| 18-24 | 75 (10.9) |
| 25-34 | 344 (50.1) |
| 35-44 | 187 (27.2) |
| >44 | 80 (11.6) |
| Gender |  |
| Female | 429 (62.2) |
| Male | 258 (37.4) |
| Non-binary | 3 (0.4) |
| Country |  |
| Canada | 150 (21.7) |
| United States | 541 (78.3) |
| Graduation year |  |
| 1971-1992 | 19 (3.2) |
| 1993-2003 | 39 (6.6) |
| 2004-2014 | 153 (25.8) |
| 2015-2023 | 358 (60.5) |
| No graduate degree | 22 (3.7) |
| Staff role |  |
| Veterinarian | 217 (32.3) |
| Licensed veterinary technician | 240 (35.8) |
| Veterinary assistant | 136 (20.3) |
| Unlicensed veterinary technician | 78 (11.6) |
| Participant dog bite history |  |
| Yes | 407 (59.2) |
| No | 230 (33.5) |
| Unsure | 50 (7.3) |
| Clinic type |  |
| Small animal practice | 361 (52.4) |
| Mixed animal practice | 209 (30.3) |
| Emergency clinic | 105 (15.2) |
| Other | 14 (2.0) |
| Stress-reducing certification |  |
| Yes | 544 (86.9) |
| No | 82 (13.1) |
| Examination room entry |  |
| Allow the dog to explore the room | 553 (80.3) |
| Immediately restraint the dog | 109 (15.8) |
| Unsure | 9 (1.3) |
| Dog approach upon entry to examination room |  |
| Approach the dog directly | 130 (19) |
| Approach the dog indirectly | 546 (79.7) |
| Unsure | 9 (1.3) |
| Response to dog struggle |  |
| Release restraint and allow the dog to calm down before re-applying restraint | 471 (68.8) |
| Apply a more restrictive restraint and/or tool | 107 (15.6) |
| Tighten grip on current restraint to prevent dog from escaping | 107 (15.6) |
