# Supplementary material for: Handling techniques and risk factors reported by veterinary professionals during dog examinations: a cross-sectional survey across Canada and the United States
Source: Front Vet Sci. 2025 Aug 18;12:1634970. doi: 10.3389/fvets.2025.1634970 (PMC12400514; doi:10.3389/fvets.2025.1634970)
Supplement: Supplementary file 2 [file Supplementary_file_1.docx]

Survey: Veterinary Staff Perceptions on Canine Handling

1. If you have read the above information and agree to participate in this study, please select the "Yes" button below.

- Yes
- No

2. Are you 18 years of age or older?

- Yes
- No

3. In what country do you currently reside?

- United States
- Canada
- Other

4. Do you conduct routine handling of canines during health examinations in a veterinary clinic setting?

- Yes
- No

**Part I: Demographic Information**

5. Are you:

- Male
- Female
- Non-binary/Third gender/Other
- Prefer not to answer

6. What is your age (in years)?

- 18-24
- 25-34
- 35-44
- 45-54
- 55-64
- 65-74
- 75 +
- Prefer not to answer

7. What is your current position?

- Veterinarian
- Licensed Veterinary Technician
- Veterinary Technician (Unlicensed)
- Veterinary Assistant
- Other (please specify) __________________________________________________

8. If you attended school for your position, what year did you graduate from your program?

▼ Not Applicable ... 1950

9. What best describes your workplace:

- Small animal practice
- Mixed animal practice
- Emergency clinic
- Other: __________________________________________________

10. What is the name of your current veterinary clinic(s):

If you prefer not to answer, please leave the box blank. This question is to account for multiple staff members from the same clinic completing the survey. To maintain anonymity, clinic name will be de-identified and not associated with your response.

________________________________________________________________

**Part II: General Handling**

11. Have you ever been bitten (teeth cut into or broke skin) during dog handling?

- Yes
- No
- Unsure
- Prefer Not to Answer

12. On average, during a routine examination, how would you respond when a dog is struggling against the physical restraint applied?

- Release the restraint and allow dog to calm down before re-applying restraint
- Apply a more restrictive restraint/tool
- Tighten grip on current restraint to prevent dog from escaping

13. Rate your level of agreement as to whether the following factors influence how you handle a dog during a routine examination:

|  | Strongly Agree | Agree | Disagree | Strongly Disagree | Prefer Not to Answer or Not Applicable |
| --- | --- | --- | --- | --- | --- |
| Veterinarian instruction |  |  |  |  |  |
| Patient behavioral history |  |  |  |  |  |
| Dog breed |  |  |  |  |  |
| Dog size |  |  |  |  |  |
| Dog age |  |  |  |  |  |
| Owner presence |  |  |  |  |  |
| Staff comfort level |  |  |  |  |  |
| Amount of time |  |  |  |  |  |

14. How often do you offer treats while handling dogs during routine veterinary examinations?

- Always
- Often
- Sometimes
- Rarely
- Never
- Prefer Not to Answer

15. Where would you typically perform the exam for a small dog and large dog?

|  | On a table with traction surface | On a table without traction surface | On the ground with traction surface | On the ground without traction surface |
| --- | --- | --- | --- | --- |
| Small dog (Less than 35 lb/16 kg) |  |  |  |  |
| Large dog (35 lb/16 kg or greater) |  |  |  |  |

16. On average, when you first enter a room for a routine veterinary examination, do you:

- Allow the dog to explore the room before handling the dog
- Immediately restrain the dog
- Unsure/Prefer not to answer

17. On average, how do you approach a dog when you first enter the room?

- Approach the dog directly (e.g., stand / walk directly towards the dog)
- Approach the dog indirectly (e.g., crouch / kneel on the ground, not facing the dog directly)
- Unsure/Prefer not to answer

**Part III: Handling Techniques**

*Please read before continuing*
 
For the next set of questions, you will be asked to indicate how often you use various dog handling techniques for routine examination on dogs when they are calm, fearful and aggressive.

The question will first be asked for small dogs (under 35 pounds/16 kilograms), then repeated for medium/large dogs (35 pounds/16 kilograms or greater). Definitions will be provided.

The questions will be set up in the following way:  

1) When a dog is **CALM** during a routine examination, how often do you use the following handling techniques?

2) When a dog is **FEARFUL** during a routine examination, how often do you use the following handling techniques?

3) When a dog is **AGGRESSIVE** during a routine examination, how often do you use the following handling techniques?

For the purposes of this study, calm, fearful, and aggressive are defined in the following ways:

| **CALM** | Relaxed, no signs of aggression or fear-related behaviors |
| --- | --- |
| **FEARFUL** | Showing fear-related behaviors, such as lowered posture, ears back, tail tucked, whimpering or whining, shaking or trembling, attempts to hide or escape |
| **AGGRESSIVE** | Showing fear-related behaviors plus baring teeth, attempting to bite, growling, lunging |

**CALM:** Relaxed, no signs of aggression or fear-related behaviors

18. When a dog is **CALM** during a routine examination, how often do you use the following handling techniques?

|  | Always | Often | | | Sometimes | Rarely | | Never | |
| --- | --- | --- | --- | --- | --- | --- | --- | --- | --- |
| **Soft muzzle**: Fabric muzzle that clips behind the ears securing mouth shut  **Small**  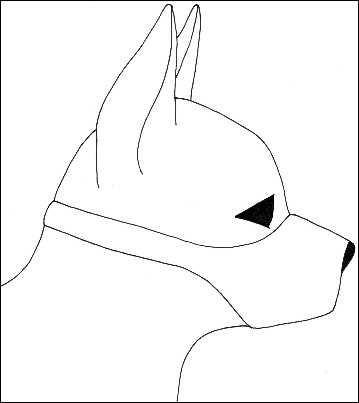 |  |  | | |  |  | |  | |
| **Medium/Large**  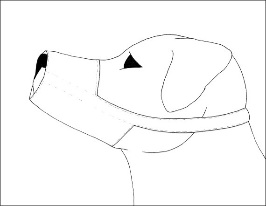 |  |  | | |  |  | |  | |
| **Muzzle hold:** Mouth held shut with both hands on muzzle  **Small**  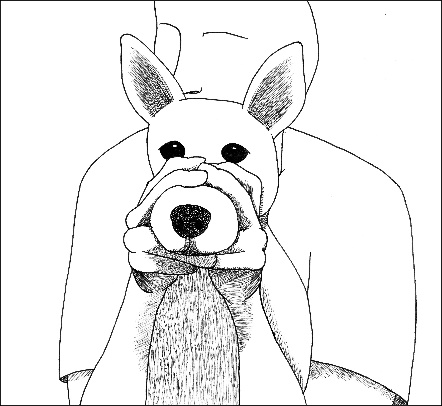 |  |  | | |  |  | |  | |
| **Medium/Large**  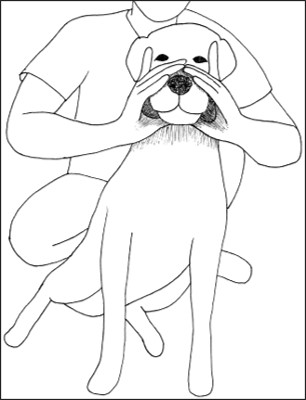 |  | |  |  | | |  | |  |
| **Towel Wrap:** Towel wrapped around the neck and held at the back to stabilize the head  **Small**  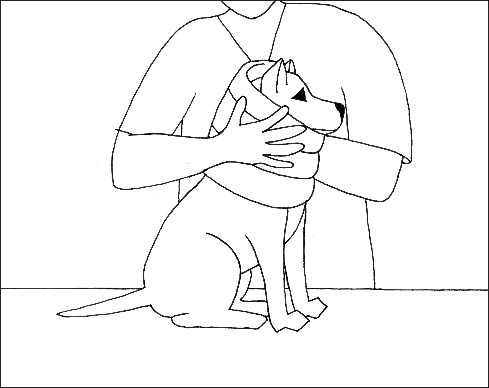 |  | |  |  | | |  | |  |
| **Medium/Large**  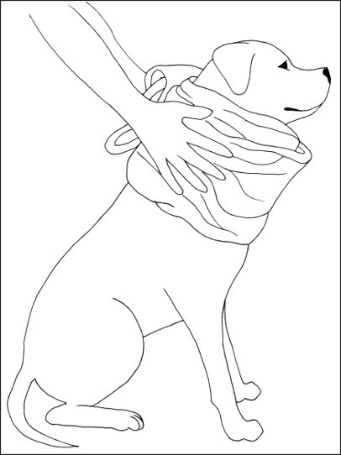 |  | |  |  | | |  | |  |
| **Minimal restraint:** Hands are placed on each side of the dog’s shoulder allowing some movement of the body and limbs  **Small**  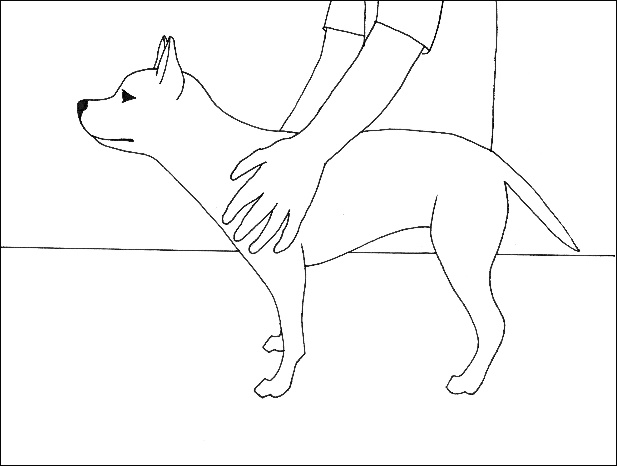 |  | |  |  | | |  | |  |
| **Medium/Large**  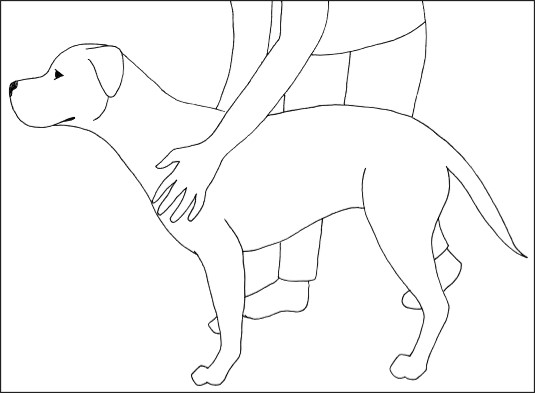 |  | |  |  | | |  | |  |
| **Secure restraint:**Hold that secures abdomen with one hand and neck with other hand  **Small**  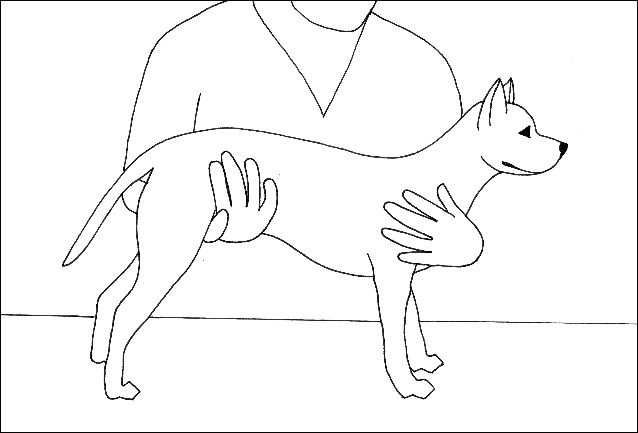 |  | |  |  | | |  | |  |
| **Medium/Large**  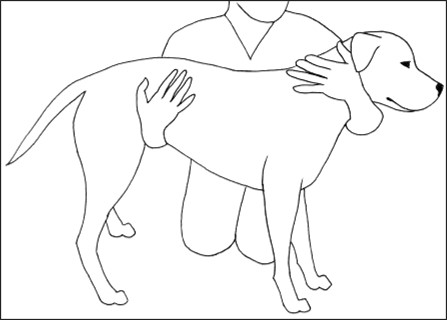 |  | |  |  | | |  | |  |
| **Head restraint (minimal):** Restraint that rests hands on either side of the dog's shoulders and abdomen  **Small**  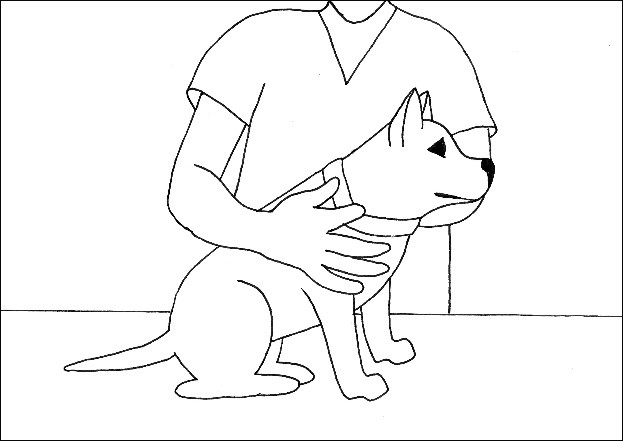 |  | |  |  | | |  | |  |
| **Medium/Large**  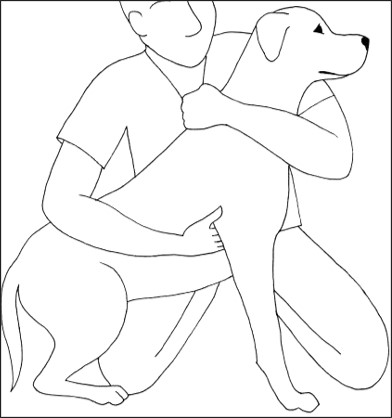 |  | |  |  | | |  | |  |
| **Head restraint (secure):**Restraint that pulls the head towards the handler's body with one hand placed around the head and an arm holding the dog's side to prevent moving  **Small**  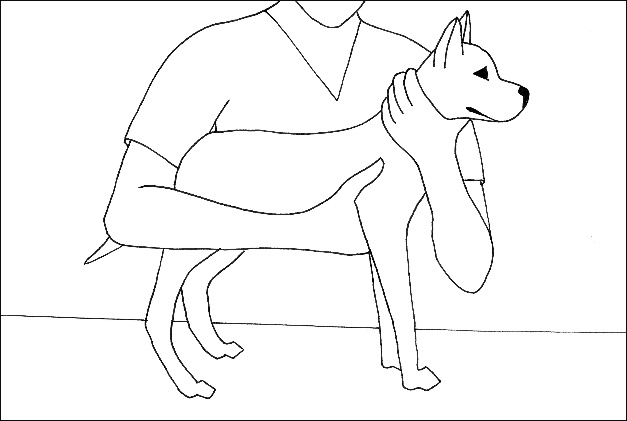 |  | |  |  | | |  | |  |
| **Medium/Large**  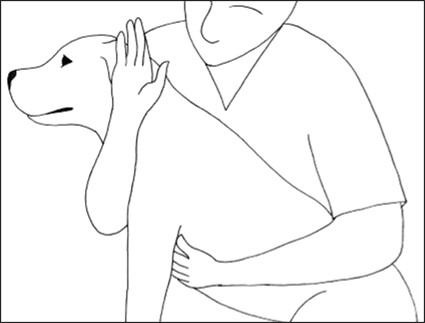 |  | |  |  | | |  | |  |
| **Laying full body restraint:**Forward leaning motion with hands secured around the neck and/or arm to prevent standing  **Small**  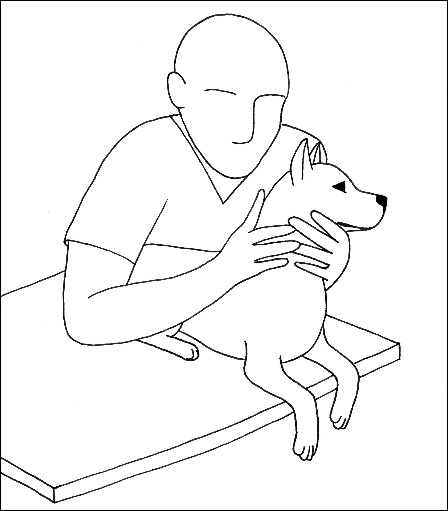 |  | |  |  | | |  | |  |
| **Medium/Large**  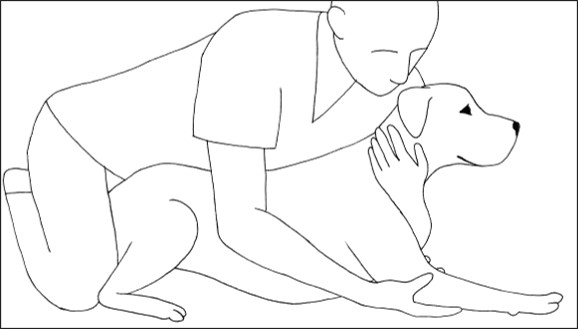 |  | |  |  | | |  | |  |
| **Full body restraint:**Restrain all legs using hands while dog is laying on side allowing little to no movement  **Small**  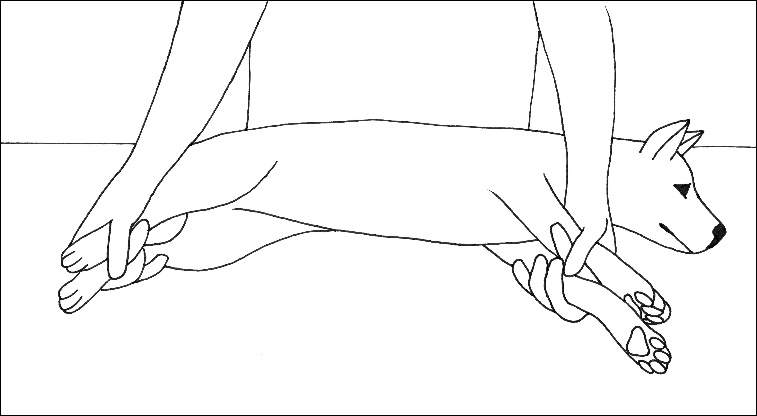 |  | |  |  | | |  | |  |
| **Medium/Large**  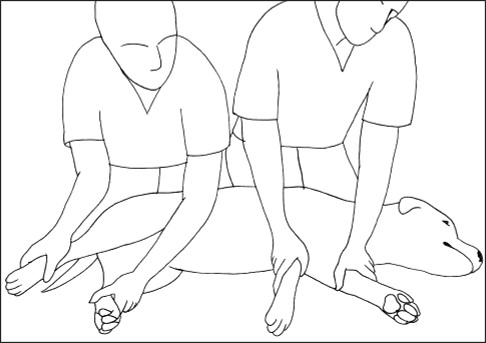 |  | |  |  | | |  | |  |
| **Sitting full body restraint:**Restraint that secures the neck and a limb with one hand while in a sitting position to prevent moving back  **Small**  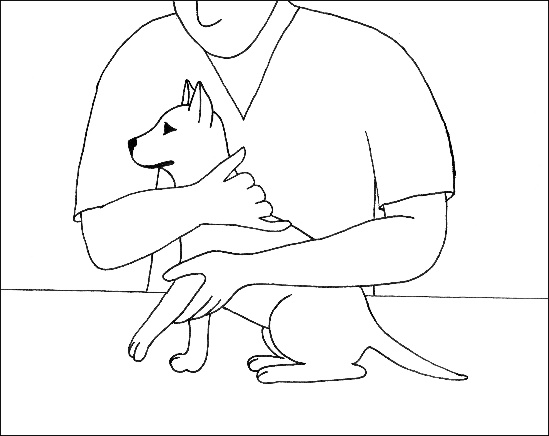 |  | |  |  | | |  | |  |
| **Medium/Large**  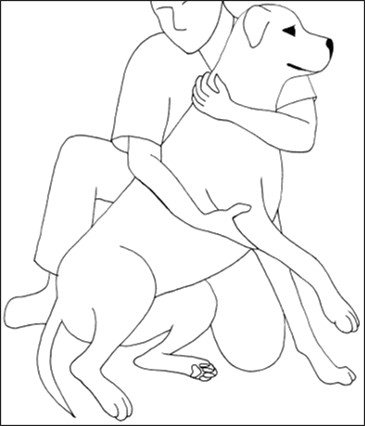 |  | |  |  | | |  | |  |
| **Elizabethan collar:** Cone shaped collar that reduces head movement and mouth access beyond collar  **Small**  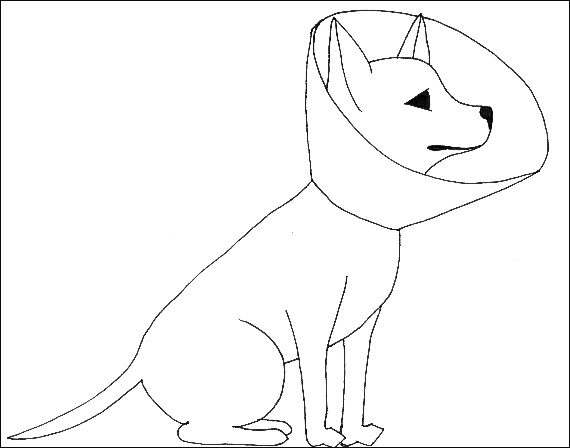 |  | |  |  | | |  | |  |
| **Medium/Large**  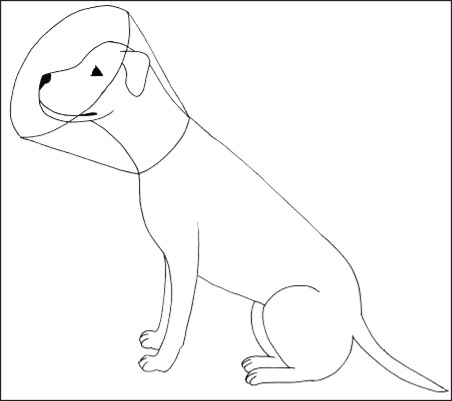 |  | |  |  | | |  | |  |
| **Dog mask:**A mask is placed over the eyes and clipped behind the head to reduce visual stimulation  **Small**  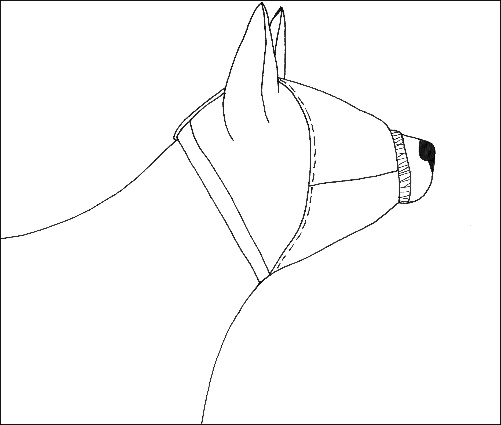 |  | |  |  | | |  | |  |
| **Medium/Large**  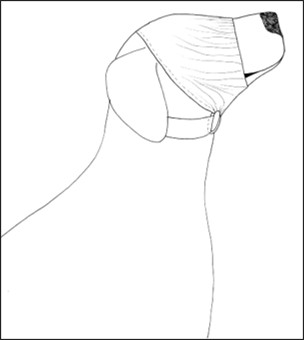 |  | |  |  | | |  | |  |
| **Basket muzzle:**Muzzle that allows for some mouth movement but limited inside the device  **Small**  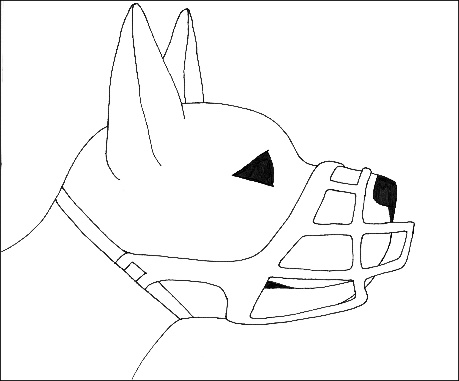 |  | |  |  | | |  | |  |
| **Medium/Large**  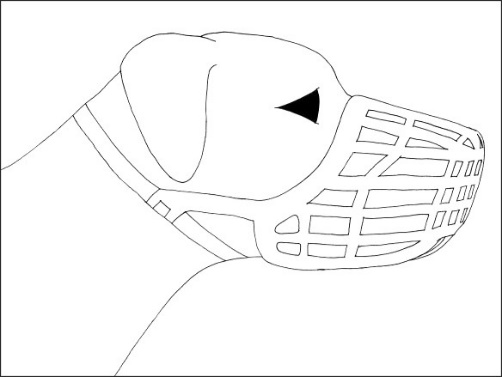 |  | |  |  | | |  | |  |
| **Chemical restraint:** sedative or anesthesia.  **Small** |  | |  |  | | |  | |  |
| **Medium/Large** |  | |  |  | | |  | |  |

**Note: The above table was repeated for when a dog is FEARFUL and AGGRESSIVE.**

**Part IV: Clinic Experience**

21. Please select which of the following courses or certification programs you have completed:

|  | Yes | No |
| --- | --- | --- |
| Fear Free Certified Veterinary Professional |  |  |
| Sophia Yin Low Stress Handling Silver Certified |  |  |
| Karen Pryor Better Veterinary Visits |  |  |
| Other, please specify: |  |  |

22. Please rank the following items from **most to least important** when completing a routine veterinary examination on a dog: **(1 = most important, 5 = least important)**

______ Completing the appointment on time

______ Client satisfaction

______ Staff safety

______ Minimizing stress for the canine patient

______ Completing all components of the examination (e.g., checking TPR, etc.)

**Part V: Professional Quality of Life (ProQOL)**

23. When you work as a veterinary professional, you have direct contact with your clients' and patient's lives. As you may have found, your compassion for those you help can affect you in positive and negative ways. Below are some questions about your experiences, both positive and negative, as a veterinary professional. Consider each of the following questions about you and your current work situation. Select the number that honestly reflects how frequently you experienced these things in the **last 30 days**.

|  | 1 = Never | 2 = Rarely | 3 = Sometimes | 4 = Often | 5 = Very Often |
| --- | --- | --- | --- | --- | --- |
| 1. I am happy. |  |  |  |  |  |
| 2. I am preoccupied with more than one person I help. |  |  |  |  |  |
| 3. I get satisfaction from being able to help people. |  |  |  |  |  |
| 4. I feel connected to others. |  |  |  |  |  |
| 5. I jump or am startled by unexpected sounds. |  |  |  |  |  |
| 6. I feel invigorated after working with those I provide care for. |  |  |  |  |  |
| 7. I find it difficult to separate my personal life from my life as a care provider. |  |  |  |  |  |
| 8. I am not as productive at work because I am losing sleep over traumatic experiences of a patient/client that I provide care for. |  |  |  |  |  |
| 9. I think that I might have been affected by the traumatic stress of those I provide care for. |  |  |  |  |  |
| 10. I feel trapped by my job as a care provider. |  |  |  |  |  |
| 11. Because of my role as a care provider, I have felt "on edge" about various things. |  |  |  |  |  |
| 12. I like my work as a care provider. |  |  |  |  |  |
| 13. I feel depressed because of the traumatic experiences of the patients/clients I provide care for. |  |  |  |  |  |
| 14. I feel as though I am experiencing the trauma of a patient/client I have provided care for. |  |  |  |  |  |
| 15. I have beliefs that sustain me. |  |  |  |  |  |
| 16. I am pleased with how I am able to keep up with care provider techniques and protocols. |  |  |  |  |  |
| 17. I am the person I always wanted to be. |  |  |  |  |  |
| 18. My work makes me feel satisfied. |  |  |  |  |  |
| 19. I feel worn out because of my work as a care provider. |  |  |  |  |  |
| 20. I have happy thoughts and feelings about those I provide care for and how I could help them. |  |  |  |  |  |
| 21. I feel overwhelmed because my case workload seems endless. |  |  |  |  |  |
| 22. I believe I can make a difference through my work. |  |  |  |  |  |
| 23. I avoid certain activities or situations because they remind me of frightening experiences of the people I patients/clients I help. |  |  |  |  |  |
| 24. I am proud of what I can do to provide care. |  |  |  |  |  |
| 25. As a result of my care, I have intrusive, frightening thoughts. |  |  |  |  |  |
| 26.I feel "bogged down" by the system. |  |  |  |  |  |
| 27. I have thoughts that I am a "success" as a care provider. |  |  |  |  |  |
| 28. I can't recall important parts of my work with trauma victims. |  |  |  |  |  |
| 29. I am a very caring person. |  |  |  |  |  |
| 30. I am happy that I chose to do this work. |  |  |  |  |  |

**Part VI: Ten Item Personality Index (TIPI)**

24. The following questions are going to ask about how you see yourself and for you to reflect on your role in the veterinary field.

I see myself as:

|  | Disagree strongly | Disagree moderately | Disagree a little | Neither agree nor disagree | Agree a little | Agree moderately | Agree strongly |
| --- | --- | --- | --- | --- | --- | --- | --- |
| 1. Extraverted, enthusiastic |  |  |  |  |  |  |  |
| 2. Critical, quarrelsome |  |  |  |  |  |  |  |
| 3. Dependable, self-disciplined |  |  |  |  |  |  |  |
| 4. Anxious, easily upset |  |  |  |  |  |  |  |
| 5. Open to new experiences, complex |  |  |  |  |  |  |  |
| 6. Reserved, quiet |  |  |  |  |  |  |  |
| 7. Sympathetic, warm |  |  |  |  |  |  |  |
| 8. Disorganized, careless |  |  |  |  |  |  |  |
| 9. Calm, emotionally stable |  |  |  |  |  |  |  |
| 10. Conventional, uncreative |  |  |  |  |  |  |  |

You did it!  You made it to the end of the survey!

25. How did you hear about this survey?

- Facebook
- Instagram
- Email
- TechAnnounce
- Family or friend
- Flyer
- Other __________________________________________________

26. Thank you for participating! If you have any additional comments or feedback, please let us know below.

________________________________________________________________

Canadian participants: For urgent needs, the Mental Health Commission of Canada has a list of crisis lines available in all Canadian provinces and territories (http://suicideprevention.ca/need-help/). For inquiries of a less urgent nature, the Canadian Mental Health Association also provides information on how one can seek help across Canada (https://cmha.ca/find-help/).

American participants: For urgent needs, call 988 or contact the National Suicide Prevention Lifeline’s Crisis Chat team. You can access the Lifeline Chat and Text (https://988lifeline.org/chat/). For inquiries of a less urgent nature, the AVMA also provides information here on how one can seek help across the United States (https://www.avma.org/resources-tools/wellbeing/get-help).

Please proceed to the next page to submit your answers and have the opportunity to enter in the gift card draw.
